# Supplementary material for: Spatially revealed roles for lncRNAs in Drosophila spermatogenesis, Y chromosome function and evolution
Source: Nat Commun. 2024 May 7;15:3806. doi: 10.1038/s41467-024-47346-w (PMC11076287; doi:10.1038/s41467-024-47346-w)
Supplement: Supplementary file 1 — Supplementary Information [file 41467_2024_47346_MOESM1_ESM.pdf]

SUPPLEMENTAL FIGURES AND TABLES

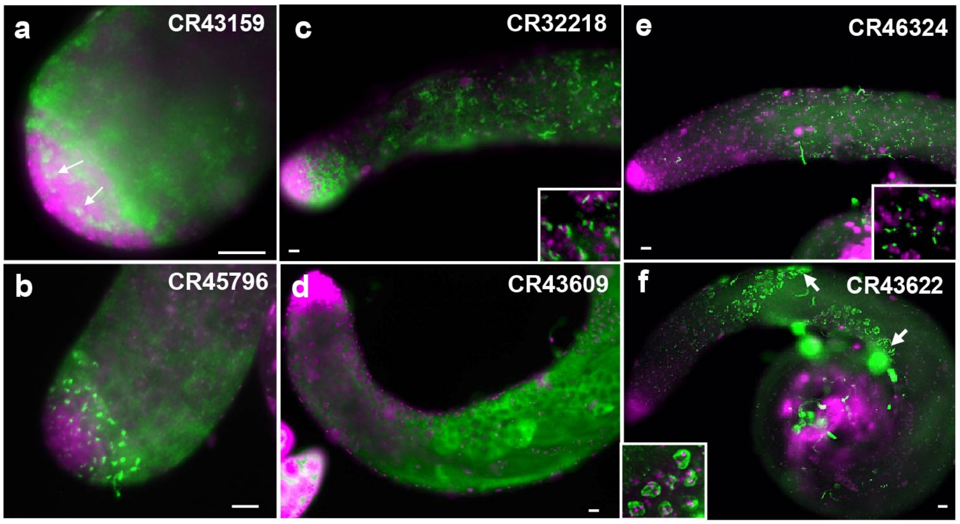

Supplemental Figure 1. lncRNA expression in the early germline. a) Early membrane associated *CR43159* lncRNA expression in germline stem and/or daughter cells, decreasing and less localized in spermatocytes (arrows indicate GSC/daughter cell interface). **b)** *CR45796* expression primarily in spermatogonia nuclei and then decreasing in early spermatocyte cytoplasm. **c)** Cytoplasmic localization of *CR32218* in spermatogonia and early spermatocytes, then decreasing and relocalized to chromatin during spermatocyte meiotic division (inset). **d)** Cytoplasmic *CR43609* expression in late spermatocytes. **e)** Increasing numbers of *CR46324* nuclear foci in spermatocyte nuclei. **f)** ‘Y-loop’ expression of *CR43622* in spermatocyte nuclei. Scale bars = 20 µm.

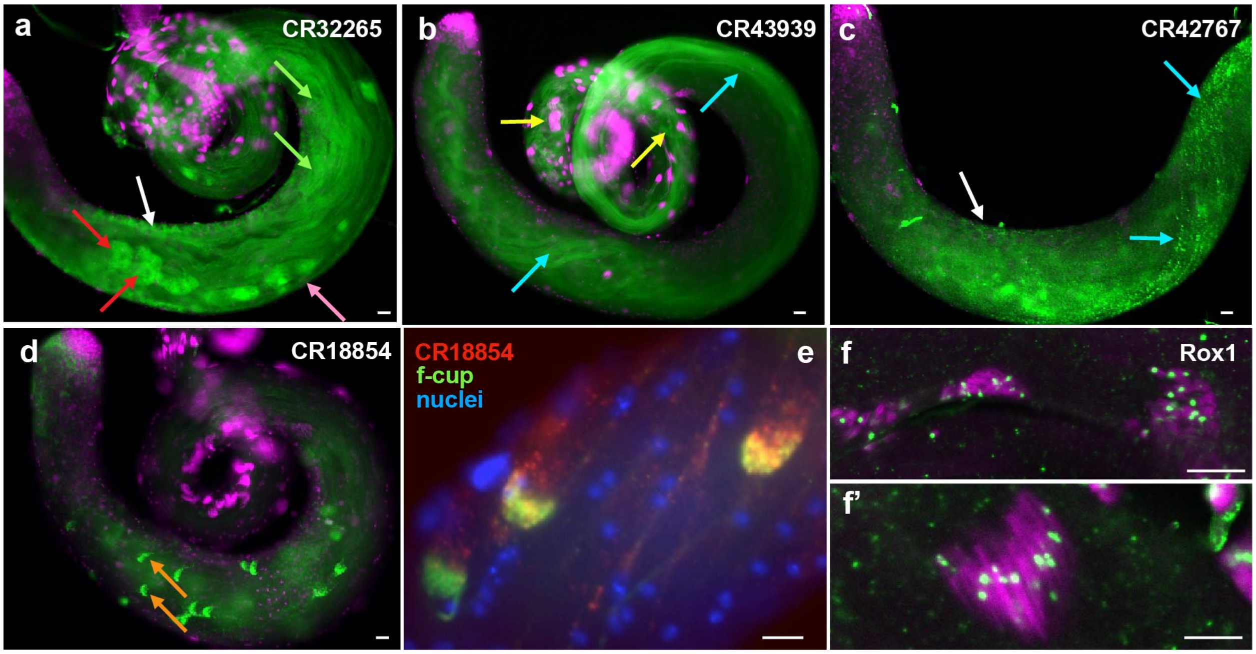

Supplemental Figure 2. Post-meiotic germline expression patterns. Panels focus on germline lncRNA expression patterns progressing from earlier to later post-meiotic stages. **a)** *CR32265* expression in round spermatids (white arrow), early elongating spermatids (green arrows), cystic bulge (pink arrow) and waste bags (red arrows). **b)** Spermatid tail expression of *CR43934* in elongated (blue arrows) and coiling spermatids (yellow arrows). **c)** Expression of *CR42767* in puncta along the outside of elongated spermatid tails (blue arrows). White arrow indicates cytoplasmic expression in round spermatids. **d)** Localization of *hpRNA: CR18854* at the ends of elongating spermatid tails (orange arrows). **e)** Double-labeling showing *CR18854* (red) co-localizing with the known cup-localized mRNA *f-cup* (green). DNA stained with DAPI (blue). **f)** Single foci likely representing nascent transcripts of *Rox1* in early elongating and **f'** elongated spermatid nuclei. In a-d, f, f': RNA (green), DNA (magenta). Scale bars = 20 μm.

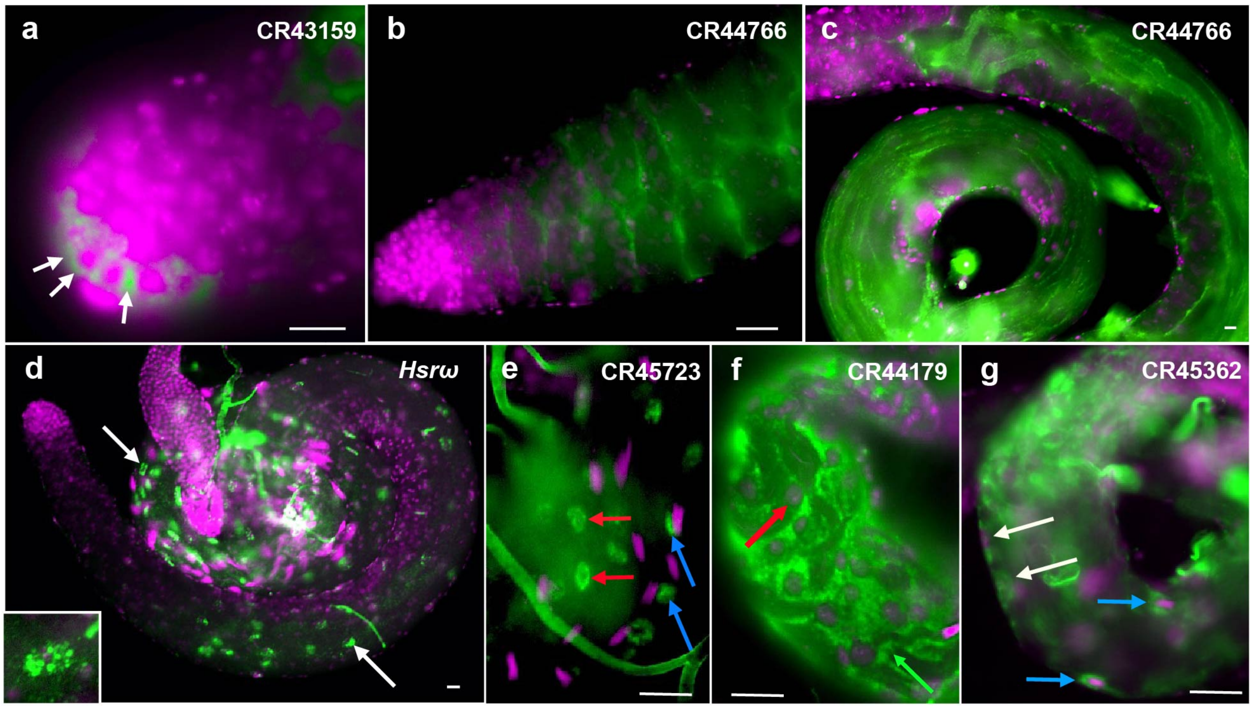

Supplemental Figure 3. lncRNA expression in cyst cells. a) *CR43159* expression in cyst stem cells. **b)** *CR44766* expression in spermatocyte-associated cyst cells. **c)** Expression of *CR44766* in cyst cells surrounding round, elongating and individualizing spermatids. **d)** *Hsrω* (*CR31400*) accumulation in puncta within cyst cell nuclei. Inset shows a closeup of a single nucleus. **e)** Nuclear localization of *CR45723* in coiling stage head (surrounding spermatid nuclear bundles; blue arrows) and tail (red arrows) cyst cells. **f)** Coiling stage cytoplasmic expression of *CR44179* primarily in tail cyst cells. Red arrows indicate nuclei at spermatid distal ends and green arrows indicate the opposite ends abutting head cyst cells (see diagram in Fig. 1e). **g)** Cytoplasmic expression of *CR45362* in coiling stage head cyst cells (blue arrows) surrounding spermatid nuclear bundles, as well as cytoplasmic expression in terminal epithelial cells (white arrows). Scale bars = 20 μm.

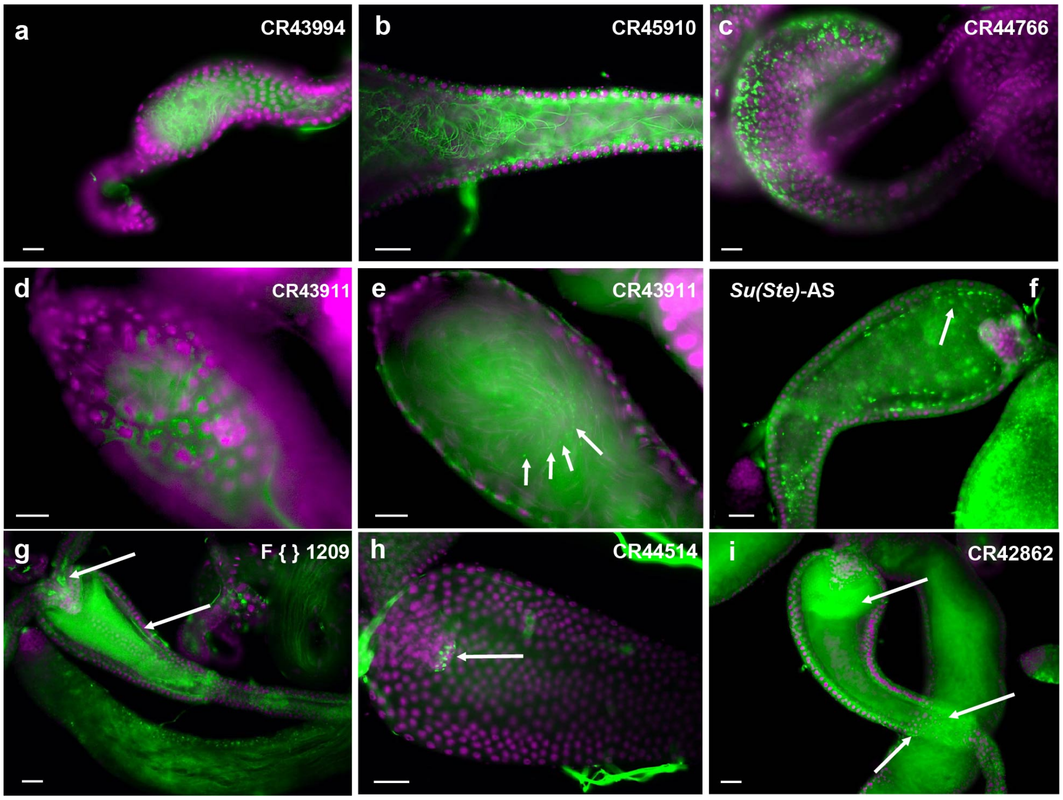

Supplemental Figure 4. Examples of lncRNA expression in seminal vesicles and ejaculatory ducts. a) *CR43994* transcripts localized within the seminal vesicle lumen. Much of the signal appears fibrous, perhaps coating mature sperm tails. **b)** *CR45910* transcripts detected as cytoplasmic puncta in seminal vesical epithelial cytoplasm and coating mature sperm tails. **c)** *CR44766* localized perinuclearly in a subset of seminal vesicle epithelial cells. **d)** *CR43911* expressed in perinuclear cytoplasmic puncta in a subset of seminal vesicle cells. **e)** *CR43911* localized in strings of puncta in the seminal vesicle lumen (arrows). *CR43911* is located just downstream of the sex peptide gene and is also secreted into the accessory gland lumen. **f)** Localization of *Su(Ste)-AS* transcripts in regularly spaced clumps at the edge of the ejaculatory duct lumen (arrow). **g)** *F-element 1209* expression in the ejaculatory duct. Transcripts are present in apical epithelial cell cytoplasm and at the junctions with accessory glands (arrows). **h)** *CR44514* expression in a ring of cells around the entrance to the ejaculatory duct lumen (arrow). **i)** Varying subcellular localization of *CR42862* in ejaculatory duct epithelial cell cytoplasm and lumen, including luminal enrichment near the duct entrance and varying basal and apical enrichment further down (arrows). Scale bars = 20 μm.

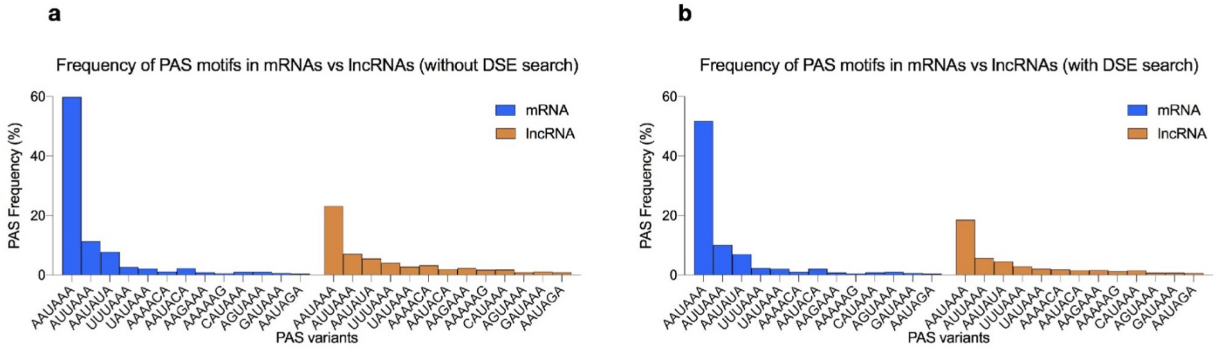

Supplemental Figure 5. Presence of polyadenylation sequences in coding and lncRNA genes. **a)** Frequency of presence of shown consensus and degenerate PAS sequences at coding and lncRNA gene 3'ends. **b)** Frequency of presence of shown consensus and degenerate PAS sequences at coding and lncRNA gene 3'ends when a potential downstream sequence element (DSE) was also detected.

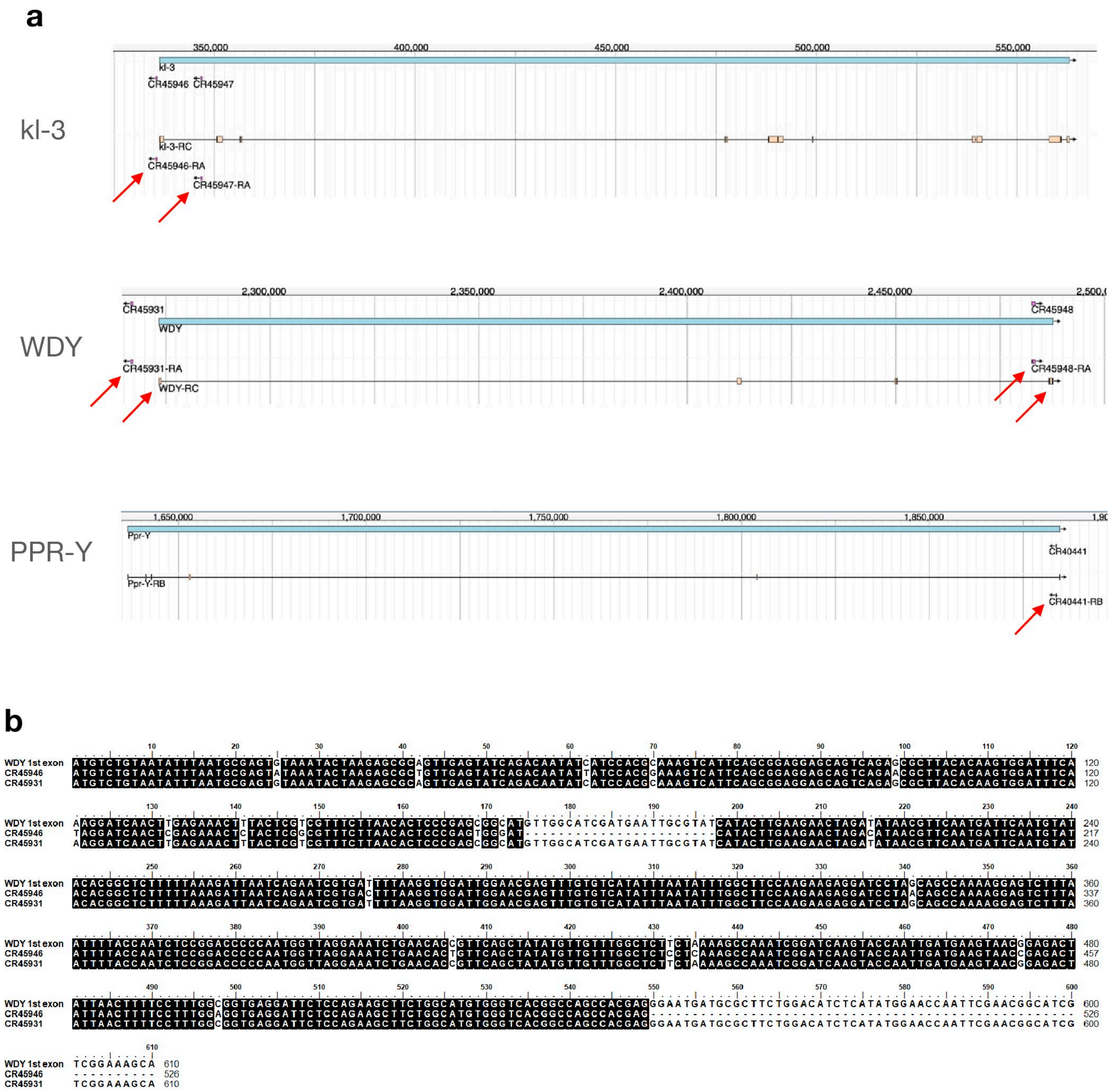

Supplemental Figure 6. Y chromosome Y-loop lncRNAs. a) Screen shot from FlyBase genome browser[63] showing the relative locations of three Y chromosome mega-genes and proximal/overlapping lncRNAs. **b)** Sequence alignments of two lncRNAs with extensive similarity to each other and to *WDY*.

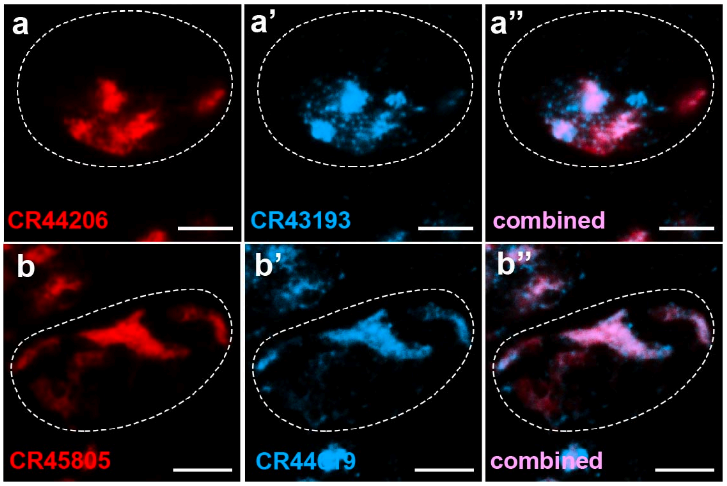

Supplemental Figure 7. Y-loop lncRNA and mega-gene co-localization. a) HCR FISH co-localization of *CR44206* and predicted lncRNA interactor *CR43193.* **b)** HCR FISH co-localization of predicted *CR44206* interactors *CR45805* and *CR44619.* Scale bars = 5 µm.

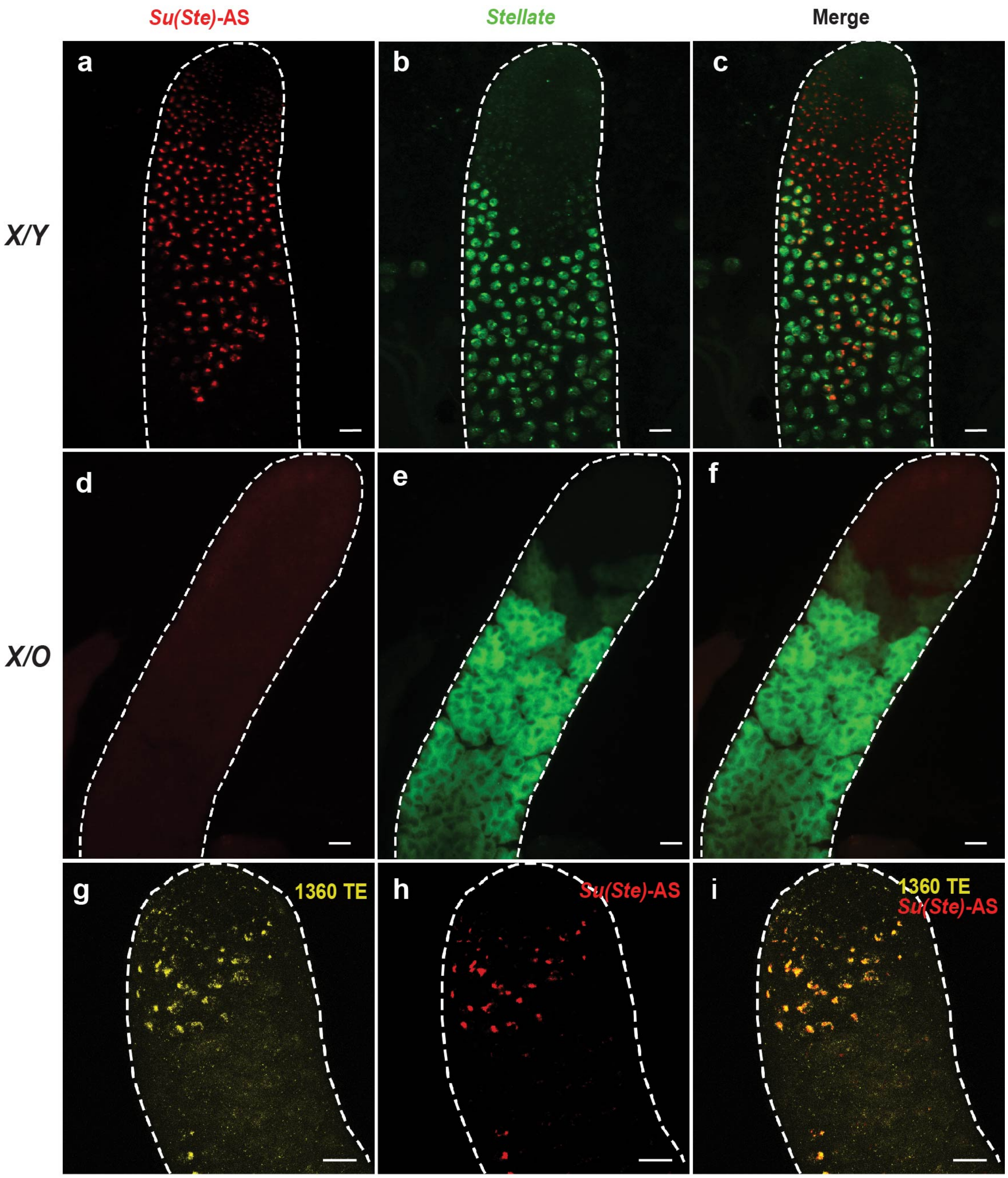

Supplemental Figure 8. ***Su*(*Ste*)-AS expression details and controls. a-c)** XY testes express both *Su(Ste)*-AS and Ste. **d-f)** Absence of *Su(Ste)*-AS transcripts (red) and increased cytoplasmic expression of *Ste* transcripts (green) in testes dissected from XO (no Y chromosome) males. Probes directed against the *hoppel/1360* TE and downstream regions of the *Su(Ste)-AS* transcript generate nearly identical patterns. Scale bars = 20 µm.

Supplemental Table 1. LncRNA genes 3' end sequences and gene-specific primers used for RT-PCR in Figure 1h.

	Gene	Sequence
1	CR32690	CATCCCAAAGAATAAAAACATCGATTAATTGAGCT
		TCAACGATCCAAATCAACGCA
2	CR42767	CATCGCAAAGAATAAAAACATCGATCAGTTGAACT
		TTCGGTGTCTTCAGTGTGCG
3	CR42850	TATAAACCAATAATAAAATATAAATTTGTATATGC
		TGGCTAACTGATGGCTGGTG
4	CR9284	TCTACGTTGATTAATTAAAGACTTTAATAAGCGTC
		CGAATCCTAGTCAATCGTTATCC
5	CR43634	AACTATTAAATACTGAATTTATAAACGATACTCGT
		TCTTTCCAAGCTAAGCGAAAT
6	CR43152	GCCCAAAAATATAAAATAAATGTTACTCCGATCTG
		TGCACTCGAAACTAAACTGGGA
7	CR45535	ACACAATCCCATTCAGTGATACTGATTGATACTTC
		TTTGTATACCAGATGGGGTGCAGTTA
8	CR45956	CGGGATTTACTACATGATGCTCCAAAGTCGACCTG
		AATCAGATGCGGGCACTAAGCATCT
9	CR44206	GAATCGACAGGAATTCGGCGAGTCAAAGAGGAGCT
		AGAGAATGATAAGGGTACGAATGCA
10	CR34044	TAATTTTGAGCATGGATCTGTTTTGGGCTGCCACT
		AATCTACGGATTAGACTGCACCTCT
11	CR43961	TTGGCCATACCGGATAACTAAATCTTTTAGCTAGC
		TTATCCGGTATGGCCAAGTTGA
12	CR44264	TCACTTAATTAACAACGGAATTTTTTATACAAGAT
		TCATGCTGACCCCAAGTTGT

PAS motifs, where present, are underlined. Genes 1-6 were chosen from genes found to be highly expressed by RNAseq and snSEQ, while genes 7-12 were chosen from lncRNA genes previously deemed very low or not detected. All 12 lncRNAs yielded strong signals via FISH analyses (Fly-FISH). Gene-specific primers used for reverse transcription are also provided.

Supplemental Table 2. Oligonucleotide sequences used for HCR FISH.

Gene	Target site	Initiator	start positions	Split_probe_1	Split_probe_2
CR44206	1st exon	B1	1	GAGGAGGGCAGCAAACGGAAGCGAGTTCTGCAGCGAAATGAATCC	TTCAGCCGCTCAATCAACCTGACACTAGAAGAGTCTTCCTTTACG
CR44206	1st intron	B2	1809	CCTCGTAAATCCTCATCAAACTGTGTTCTGTGTTCCTCGGTGCTC	TGTGTTCTGAGTTCTGCGTTCTGTGAAATCATCCAGTAAACCGCC
CR44206	3rd exon	B3	15406	GTCCCTGCCTCTATATCTTTGTCGATTCAATGGGTATTGGTTTGA	AGCTCCTCTTTGACTCGCCGAATTCTTCCACTCAACTTTAACCCG
CR44619	1st exon	B2	140	CCTCGTAAATCCTCATCAAACTCGCTCTCTCTTGCCCCTTTTACG	TCATGTTAACTGGTGAGCTCTCCCTAAATCATCCAGTAAACCGCC
CR45805	N	B1	89	GAGGAGGGCAGCAAACGGAACTCTCTCTTCAAAAAGAGCGTGCGC	TCTCTCTCTCTCTCTCTCTCTCTCATAGAAGAGTCTTCCTTTACG
CR43193	exon/1st intron junc	B1	558	GAGGAGGGCAGCAAACGGAAACGTGCAAGCTGCATGAATTACGAG	TTTTCCCCGGACCTCTGCTCTTTTGTAGAAGAGTCTTCCTTTACG
CR45931	N	B1	550	GAGGAGGGCAGCAAACGGAAGAGATGTCCAGAAGCGCATCATTCC	ACGATGCCGTTCGAATTGGTTCCATTAGAAGAGTCTTCCTTTACG
CR45931	N	B2	550	CCTCGTAAATCCTCATCAAAGAGATGTCCAGAAGCGCATCATTCC	ACGATGCCGTTCGAATTGGTTCCATAAATCATCCAGTAAACCGCC
Ppr-y	5th exon	B1	167702	GAGGAGGGCAGCAAACGGAATTCTCGCTCATCTAATCGCAAAGTG	TTCCTTTGTTCGAGTCGCAATTGTGTAGAAGAGTCTTCCTTTACG
Ppr-y	6th exon	B1	248273	GAGGAGGGCAGCAAACGGAACGGTCATCATTTCTACAAAGAAGTT	CATCGTGTGGTAAGGATGTCATAGCTAGAAGAGTCTTCCTTTACG
Ppr-y	1st exon	B1	17	GAGGAGGGCAGCAAACGGAAGGCTTCTGTACTTCGGAATTATTTA	TCTGGGTAAATAACTTCATCCACAATAGAAGAGTCTTCCTTTACG
Ppr-y	1st exon	B1	85	GAGGAGGGCAGCAAACGGAAAGCTTTCAATCAAGGAACGGTTAAT	CACCACGATGAACGTGTTTTAAATATAGAAGAGTCTTCCTTTACG
kl-3	1st exon	B2	150	CCTCGTAAATCCTCATCAAAGCAGCACGCTTTAACATGTTGCGCG	TCTTGGTCACTTACACTAGGTCTCCAAATCATCCAGTAAACCGCC
kl-3	1st exon	B2	976	CCTCGTAAATCCTCATCAAACAAAGGTCCTACATCGGGTGTATCT	CATACGCCGCCAATTGACCAACATTAAATCATCCAGTAAACCGCC
kl-3	1st intron	B3	9255	GTCCCTGCCTCTATATCTTTAACATCCTGCTATCCGCATTCGAGA	ATACTCACCAGGATGAATGGCAAGATTCCACTCAACTTTAACCCG
kl-3	1st intron	B3	8853	GTCCCTGCCTCTATATCTTTAAGTTAAGATCGGACCCGACCCTTA	AAGCTTACGTACGCAGAGATGCTTATTCCACTCAACTTTAACCCG
Ste	2nd exon	B1	N/A	TCGGCAGCATCGAGAAGAAGATGTCAAATCATCCAGTAAACCGCC	CCTCGTAAATCCTCATCAAACGGGAAGCTGGGCCCGAACATCGCT
Ste	2nd exon	B1	N/A	CGTCCAGCTGTGTATCAGACTTCGGAAATCATCCAGTAAACCGCC	CCTCGTAAATCCTCATCAAAATGAAAGTTGCTTTTACACCGTGGG
Ste	2nd exon	B1	N/A	AGTGGATCTTGACGGTTGACTTGCCAAATCATCCAGTAAACCGCC	CCTCGTAAATCCTCATCAAACCATACAGCGCTGAGGCCCACTGGC
Ste	2nd exon	B1	N/A	GGGTGTTCTGCCTATCACAGGAGATAAATCATCCAGTAAACCGCC	CCTCGTAAATCCTCATCAAAATTGGGACACGATCCAAAATCTCCT
Ste	2nd exon	B1	N/A	GCAAATATTTTCGGTGCATAGCAATAAATCATCCAGTAAACCGCC	CCTCGTAAATCCTCATCAAACAGGCCACGCTCTGACCTGATGTAT
Ste	2nd exon	B1	N/A	GGGCGTGAATCATGCCGTACCACTTAAATCATCCAGTAAACCGCC	CCTCGTAAATCCTCATCAAACTTTTCATCGCCGTACAACAAGCCA
Ste	2nd exon	B1	N/A	AGGAACTATCGATCACCGGCTTCAGAAATCATCCAGTAAACCGCC	CCTCGTAAATCCTCATCAAAGATCACGTCCAGTATCTCGCTGAAA
Ste	2nd exon	B1	N/A	ACTCCAAGCCCATCTGGTTGAACGTAAATCATCCAGTAAACCGCC	CCTCGTAAATCCTCATCAAAATCCTGCACGTAGTCGGTGGGCACG
Su(Ste)-S	Y-chr specific region	B2	N/A	CCTCGTAAATCCTCATCAAAGAACATGGCTGTGTATCAGTTTTAG	AGAAGATGTCCGGGAAGCTGGGCTCAAATCATCCAGTAAACCGCC
Su(Ste)-S	Y-chr specific region	B2	N/A	CCTCGTAAATCCTCATCAAACGGTCTCAAGTTCGGCAGCTGCGTT	TTACTTACCGTGGGTCGTCCAGGGGAAATCATCCAGTAAACCGCC
Su(Ste)-S	Y-chr specific region	B2	N/A	CCTCGTAAATCCTCATCAAAAAGAACATTGAAGCGCTTGACTTCG	AGTTAAAAAATCACGTCATCCCTAGAAATCATCCAGTAAACCGCC
Su(Ste)-AS	Y-chr specific region	B3	N/A	GTCCCTGCCTCTATATCTTTGGTAATCACCTGGGTATAATAATAA	GCAAAGCATACTTTTCGTTAATAGTTTCCACTCAACTTTAACCCG
Su(Ste)-AS	Y-chr specific region	B3	N/A	GTCCCTGCCTCTATATCTTTGGTCATATCAAGCACTCATTCGAGT	TGGTTCAAAGTGTTCGGTCCAAGCTTTCCACTCAACTTTAACCCG

Each line contains two sequences (split probe 1/2) that target adjacent gene sequences to generate amplified signals. Target sites and starting positions on target sequences are also indicated, along with HCR initiator sequences used for amplification.
