# Supplementary material for: Spatially revealed roles for lncRNAs in Drosophila spermatogenesis, Y chromosome function and evolution
Source: Nat Commun. 2024 May 7;15:3806. doi: 10.1038/s41467-024-47346-w (PMC11076287; doi:10.1038/s41467-024-47346-w)
Supplement: Supplementary file 3 — Reporting Summary [file 41467_2024_47346_MOESM3_ESM.pdf]

# nature portfolio

Corresponding author(s): Henry M. Krause

Last updated by author(s): Jun 12, 2023



# Reporting Summary

Nature Portfolio wishes to improve the reproducibility of the work that we publish. This form provides structure for consistency and transparency in reporting. For further information on Nature Portfolio policies, see our Editorial Policies and the Editorial Policy Checklist.

## Statistics

For all statistical analyses, confirm that the following items are present in the figure legend, table legend, main text, or Methods section.

| n/a | Confirmed | |
|---|---|---|
| ☐ | ☒ | The exact sample size (*n*) for each experimental group/condition, given as a discrete number and unit of measurement |
| ☒ | ☐ | A statement on whether measurements were taken from distinct samples or whether the same sample was measured repeatedly |
| ☒ | ☐ | The statistical test(s) used AND whether they are one- or two-sided<br>*Only common tests should be described solely by name; describe more complex techniques in the Methods section.* |
| ☒ | ☐ | A description of all covariates tested |
| ☒ | ☐ | A description of any assumptions or corrections, such as tests of normality and adjustment for multiple comparisons |
| ☒ | ☐ | A full description of the statistical parameters including central tendency (e.g. means) or other basic estimates (e.g. regression coefficient) AND variation (e.g. standard deviation) or associated estimates of uncertainty (e.g. confidence intervals) |
| ☒ | ☐ | For null hypothesis testing, the test statistic (e.g. *F*, *t*, *r*) with confidence intervals, effect sizes, degrees of freedom and *P* value noted<br>*Give P values as exact values whenever suitable.* |
| ☒ | ☐ | For Bayesian analysis, information on the choice of priors and Markov chain Monte Carlo settings |
| ☒ | ☐ | For hierarchical and complex designs, identification of the appropriate level for tests and full reporting of outcomes |
| ☒ | ☐ | Estimates of effect sizes (e.g. Cohen's *d*, Pearson's *r*), indicating how they were calculated |

*Our web collection on statistics for biologists contains articles on many of the points above.*

## Software and code

Policy information about availability of computer code

| | |
|---|---|
| Data collection | No software programs were created or used for our analyses |
| Data analysis | No data anlysis programs were created or used for our analyses |

For manuscripts utilizing custom algorithms or software that are central to the research but not yet described in published literature, software must be made available to editors and reviewers. We strongly encourage code deposition in a community repository (e.g. GitHub). See the Nature Portfolio guidelines for submitting code & software for further information.

## Data

Policy information about availability of data

All manuscripts must include a data availability statement. This statement should provide the following information, where applicable:

- Accession codes, unique identifiers, or web links for publicly available datasets
- A description of any restrictions on data availability
- For clinical datasets or third party data, please ensure that the statement adheres to our policy

All in situ images acquired, as well as corresponding annotations, can be accessed on our Fly-FISH database (https://fly-fish.ccbr.utoronto.ca/). Included search tools allow searches by gene name, localization term or combinations thereof. Gene-specific information was also obtained from the FlyBase database (http://flybase.org). This information is included in our data availability statement.

## Research involving human participants, their data, or biological material

Policy information about studies with human participants or human data. See also policy information about sex, gender (identity/presentation), and sexual orientation and race, ethnicity and racism.

| | |
|---|---|
| Reporting on sex and gender | N/A |
| Reporting on race, ethnicity, or other socially relevant groupings | N/A |
| Population characteristics | N/A |
| Recruitment | N/A |
| Ethics oversight | N/A |

Note that full information on the approval of the study protocol must also be provided in the manuscript.

# Field-specific reporting

Please select the one below that is the best fit for your research. If you are not sure, read the appropriate sections before making your selection.

☒ Life sciences ☐ Behavioural & social sciences ☐ Ecological, evolutionary & environmental sciences

For a reference copy of the document with all sections, see nature.com/documents/nr-reporting-summary-flat.pdf

# Life sciences study design

All studies must disclose on these points even when the disclosure is negative.

| | |
|---|---|
| Sample size | This study was based on analyses of over 600 annotated lncRNA genes in the Drosophila genome. There are currently ~2,500 annotated lncRNA genes. Thus, this study represents approximately 1/4 of the fly lncRNA genome. Images annotated and shown are representative of those seen in multiple samples and experimental repeats. Examples of these are provided on our FlyFISH database. |
| Data exclusions | There were no data exclusions. |
| Replication | Approximately 15% of the genes analyzed were tested twice or more with similar results. In many of these cases, probes were made against different parts of the genes, or made and tested by alternative methods. |
| Randomization | The vast majority of lncRNA genes tested were randomly chosen based on cDNA library content/availability or CR number. We also included 105 lncRNAs that had been tested previously by CRISPR for effects on male fertility. |
| Blinding | The data presented are representative of what was obtained, often by multiple lab personnel. Annotation terms were reviewed by four lab members |

# Reporting for specific materials, systems and methods

We require information from authors about some types of materials, experimental systems and methods used in many studies. Here, indicate whether each material, system or method listed is relevant to your study. If you are not sure if a list item applies to your research, read the appropriate section before selecting a response.

### Materials & experimental systems

| n/a | Involved in the study | |
|---|---|---|
| ☐ | ☒ | Antibodies |
| ☒ | ☐ | Eukaryotic cell lines |
| ☒ | ☐ | Palaeontology and archaeology |
| ☐ | ☒ | Animals and other organisms |
| ☒ | ☐ | Clinical data |
| ☒ | ☐ | Dual use research of concern |
| ☒ | ☐ | Plants |

### Methods

| n/a | Involved in the study | |
|---|---|---|
| ☒ | ☐ | ChIP-seq |
| ☒ | ☐ | Flow cytometry |
| ☒ | ☐ | MRI-based neuroimaging |

## Antibodies

| | |
|---|---|
| Antibodies used | rabbit anti-GFP (Abcam # 290), mouse anti-S5 (obtained from Harald Saumweber), Alexa488-tagged goat anti-mouse (Invitrogen #A-11001). |
| Validation | All antibodies used worked as described by the merchant or as previously reported. |

## Animals and other research organisms

Policy information about studies involving animals; ARRIVE guidelines recommended for reporting animal research, and Sex and Gender in Research

| | |
|---|---|
| Laboratory animals | The study used 2-3 days post-eclosed Drosophila melanogaster white w1118 adult male flies. |
| Wild animals | The study did not involve wild animals |
| Reporting on sex | This study only used adult male reproductive tissues. |
| Field-collected samples | No field collected samples were used in this study. |
| Ethics oversight | This study did not require ethics oversight. |

Note that full information on the approval of the study protocol must also be provided in the manuscript.
